# A Comparative Study on Physicochemical Properties and Biological Activities of Polysaccharides from *Coreopsis tinctoria* Buds Obtained by Different Methods

**DOI:** 10.3390/foods14071168

**Published:** 2025-03-27

**Authors:** Shang Gao, Weipei Li, Moupan Yin, Rui-Bo Jia, Chunxia Zhou, Xinhui Pan, Bingwu Liao

**Affiliations:** 1 Key Laboratory of Ministry of Education for Xinjiang Phytomedicine Resource and Utilization, School of Pharmacy, Shihezi University, Shihezi 832003, China; 2 College of Food Science and Technology, Guangdong Ocean University, Guangdong Provincial Key Laboratory of Aquatic Product Processing and Safety, Guangdong Province Engineering Laboratory for Marine Biological Products, Guangdong Provincial Engineering Technology Research Center of Seafood, Guangdong Provincial Engineering Technology Research Center of Prefabricated Seafood Processing and Quality Control, Zhanjiang 524088, China

**Keywords:** *Coreopsis tinctoria* bud polysaccharides, extraction method, degradation, characterization, in vitro activities

## Abstract

In this study, the polysaccharides of *Coreopsis tinctoria* buds (CTBPs) were extracted by hot water, ultrasound, alkali solution, and acid solution, and the four kinds of extracted polysaccharides were denoted Hw, Ultra, Al, and Ac. Then, the Hw were degraded by ultrasound, an alkali solution, and an acid solution, and the three resultant kinds of polysaccharides were denoted Ultra-Post-proc, Al-Post-proc, and Ac-Post-proc. The study comprehensively compared and analyzed the physical and chemical characteristics, structural properties, and in vitro activities of each polysaccharide. The extraction and treatment methods significantly affected the chemical composition, molecular weight (Mw) and potential of the CTBPs. The contents of carbohydrates, total phenol, and protein in Al were the highest, at 78.79 ± 0.62%, 81.69 ± 0.70 mg GAE/g and 4.82 ± 0.10%, respectively. The different methods did not change the monosaccharide composition of CTBPs, but affected the monosaccharide proportion and reduced the Mw of CTBPs. The absolute zeta potential value of Al was the highest, indicating that the solution was the most stable. CTBPs had the characteristic structure of polysaccharides, and Al-Post-proc had a triple helix structure. Additionally, CTBPs also had good water and oil holding abilities, as well as bile acid binding ability. CTBPs displayed good activity in vitro, among which Al possessed the best *α*-glucosidase inhibitory activity and the strongest free radical scavenging ability, and also well inhibited the generation of glycosylation products and protein oxidation products in the bovine serum albumin (BSA)-fructose model. These findings provide support for a theoretical basis for the application of polysaccharide from *Coreopsis tinctoria* bud in pharmaceutical and functional foods.

## 1. Introduction

Polysaccharides are important natural macromolecular polymers that are widely distributed in nature. They are polyhydroxy polymers formed by multiple monosaccharides of the same or different types connected by glycosidic bonds. Based on the type of monosaccharides, polysaccharides can be categorized into homopolysaccharides (consisting of one type of monosaccharide) and heteropolysaccharides (consisting of multiple types of monosaccharides) [1]. According to their sources, polysaccharides can be divided into plant, animal, microbial, and synthetic polysaccharides [2]. These polysaccharides not only fulfill the basic functions of biological energy storage and cell structural support [3], but also exhibit a variety of biological activities, including anti-inflammatory [4], anti-tumor [5], anti-oxidative [6], and hypoglycemic effects [7], as well as regulation of the gut microbiota [8] and so on. Wei et al. [9] evaluated the anti-inflammatory activity of polysaccharides extracted from *Typha angustifolia*; the purified polysaccharides exerted their anti-inflammatory effects by inhibiting the production of inflammatory factors and reactive oxygen species (ROS). *Dendrobium officinale* polysaccharides induced apoptosis of tumor cells by altering mitochondrial function, ROS production, and expression of apoptosis-related proteins [10]. Tea polysaccharides ameliorated hyperglycemia and hyperlipidemia by modulating the gut microbiota and enhancing host metabolic functions [11]. In addition, wheat bran polysaccharides exhibited hypoglycemic activity through non-competitive inhibition of *α*-glucosidase [12]. *Auricularia auricula* polysaccharides could alleviate protein oxidative damage, inhibit the formation of advanced glycosylation end products (AGEs), and ameliorate cell fibrosis [13]. A large number of studies have shown that polysaccharides exhibit favorable activities. However, the activities and structures of polysaccharides are influenced not only by different extraction methods, but also by degradation treatments. As a result, an increasing number of methods for obtaining polysaccharides are being investigated.

The traditional extraction method for polysaccharides involves hot water extraction followed by ethanol precipitation to obtain the polysaccharides. Different extraction methods for polysaccharides include ultrasonic extraction, alkaline solution extraction, acid solution extraction, and enzymatic extraction, among others. Degradation methods for polysaccharides include alkaline solution degradation, acid solution degradation, ultrasonic degradation, hydrogen peroxide degradation, and others. Compared with traditional hot water extraction, ultrasonic extraction of *Choerospondias axillaris* peel polysaccharides resulted in lower molecular weights and higher antioxidant activity in vitro [14]. By optimizing the acid degradation parameters for the seeds of *Plantago ovata Forssk* polysaccharides, Nuerxiati et al. [15] obtained a low-viscosity polysaccharide. Further purification yielded a polysaccharide with strong free radical scavenging abilities, which could inhibit the secretion of pro-inflammatory factors. In our previous study [16], the polysaccharides were further treated using water, acid, and alkali solution extract, combined with ultrasonic degradation. After ultrasonic treatment, the molecular weight (Mw) of the polysaccharide was reduced, and its in vitro activities were enhanced, including antioxidant activity and *α*-glucosidase inhibitory activity. It has been demonstrated that the polysaccharide obtained through alkali solution extraction combined with ultrasonic treatment exhibited the best activities. It can be seen that polysaccharides with different properties can be obtained by various methods, which facilitates the production of polysaccharides with high yields and high activities.

*Coreopsis tinctoria* bud, the flower bud of *Coreopsis tinctoria*, which is not fully opened, is a medicinal plant widely distributed in southern Xinjiang, China [17], it is often consumed as a kind of folk medicinal tea by the local population [18], which can play a role in lowering blood sugar and removing free radicals [17]. Studies have shown that polysaccharides are one of its active components; *Coreopsis tinctoria* polysaccharides have been mentioned in a small number of studies. The alkali-soluble polysaccharide obtained from *Coreopsis tinctoria* was isolated and purified to produce a arabinogalactan, which had the potential to treat diabetes mellitus and Alzheimer’s disease [18]. Yuan et al. [19] compared the properties of different chrysanthemum polysaccharides and found that the main monosaccharides of chrysanthemum polysaccharides are Galacturonic acid, Arabinose, and Galactose. In particular, the polysaccharides extracted from *Coreopsis tinctoria* exhibited significant free radical scavenging activities and also had strong anti-glycosylation activity. It can be seen that, at present, there are few studies on the polysaccharides of *Coreopsis tinctoria* buds; in particular, comparative studies of polysaccharides obtained by different methods are lacking. Based on this, the present study compared different methods of polysaccharide extraction and degradation. Firstly, four kinds of *Coreopsis tinctoria* bud polysaccharides (CTBPs) were obtained using hot water, ultrasound, alkali solutions, and acid solutions, and were denoted as Hw, Ultra, Al, and Ac. Then, three kinds of degraded CTBPs were obtained by ultrasound, alkali, and acid degradation of Hw, and denoted as Ultra-Post-proc, Al-Post-proc, and Ac-Post-proc. The monosaccharide composition, molecular weight, structural characteristics, oil and water holding capacities, bile acid-binding capacity, *α*-glucosidase inhibitory activity, antioxidant activity, and anti-glycosylation activity of CTBPs were compared. The influences of different extraction and degradation methods on CTBPs were comprehensively analyzed and evaluated to provide a basis for the development and utilization of CTBPs.

## 2. Materials and Methods

### 2.1. Materials and Reagents

*Coreopsis tinctoria* buds were obtained from a nearby marketplace in Dabancheng (Xinjiang Uyghur Autonomous Region, China). All monosaccharide standards, bovine serum albuminwere and fructose were obtained from Shanghai Yuanye Co., Ltd., (Shanghai, China). All other reagents used were of analytical grade.

### 2.2. Preparation of Coreopsis tinctoria Buds Polysaccharides (CTBPs)

**Figure 1:**
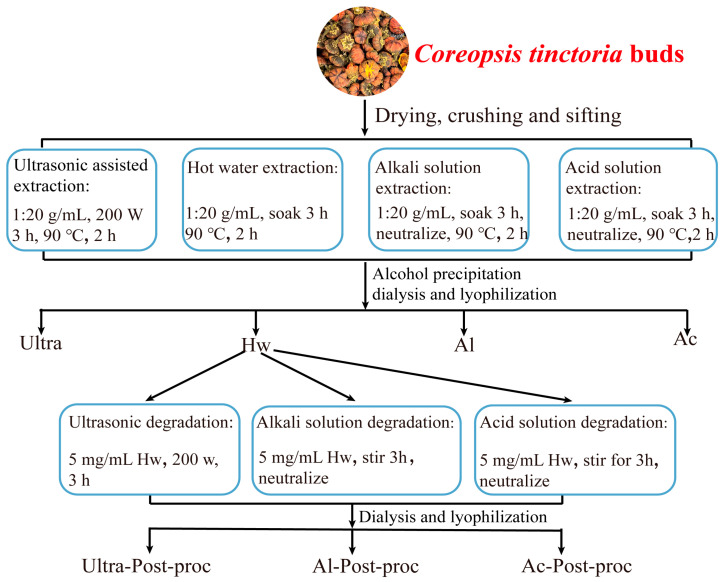
CTBPs extraction flow chart.

The dried *Coreopsis tinctoria* buds were crushed and passed through a 60-mesh sieve to prepare the powder. The powder was then stored in a sealed, dry place for subsequent use. The specific extraction methods of CTBPs are as follows, in Figure 1.

Extraction of polysaccharides using hot water: 50 g of *Coreopsis tinctoria* buds powder was precisely weighed and 1000 mL of deionized water was added. The mixture was soaked for 3 h and continuously stirred in a water bath at 90 °C for 2 h. After cooling, the mixture was centrifuged at 10,000× *g* for 10 min. The supernatant was collected through gauze filtration and concentrated to 1/3–1/4 of its original volume under reduced pressure. Subsequently, 95% ethanol was added to the concentrated solution at a volume ratio of 4:1 and allowed to stand at 4 °C for 12 h. The precipitates were collected, redissolved in deionized water, and dialyzed in a 3500 Da dialysis bag for 72 h. Finally, the solution was freeze-dried to obtain the polysaccharides extracted by hot water, and designated as Hw.

Ultrasonic-assisted extraction of polysaccharides: 50 g of powdered *Coreopsis tinctoria* buds were precisely weighed, and 1000 mL of deionized water was added. The mixture was subjected to ultrasonic treatment at a power of 200 W for 3 h. The subsequent operational steps were identical to those of the hot water extraction method. The polysaccharides obtained through ultrasonic-assisted extraction were designated as Ultra.

Extraction of polysaccharides with alkali solution: 50 g of powdered *Coreopsis tinctoria* buds were precisely weighed, and 1000 mL of 0.1 M NaOH solution added. The mixture was soaked for 3 h, after which the pH was adjusted to 7. The subsequent operational steps were identical to those of the hot water extraction method. The polysaccharides obtained through the alkali solution extraction were designated as Al.

Extraction of polysaccharides with acid solution: 50 g of powdered *Coreopsis tinctoria* buds were precisely weighed, and 1000 mL of 0.1 M HCl solution was added. The mixture was soaked for 3 h, after which the pH was adjusted to 7. The subsequent operational steps were identical to those of the hot water extraction method. The polysaccharides obtained through acid solution extraction were designated as Ac.

Ultrasonic degradation of polysaccharides: Hw was added into a 5 mg/mL solution using deionized water. Then, the solution was subjected to ultrasonic treatment in an ultrasonic cleaning instrument (KQ-300DE, Kunshan, China) at a power of 200 W for 3 h. Subsequently, the solution was vacuum-concentrated, dialyzed in a 3500 Da bag for 72 h, and underwent freeze-drying. After these steps, the polysaccharides obtained through ultrasonic degradation were designated as Ultra-Post-proc.

Alkali solution for polysaccharide degradation: Hw was added into a 5 mg/mL solution using 0.1 M NaOH solution. The solution was soaked and stirred for 3 h, after which the pH was adjusted to 7. Then, the solution was vacuum-concentrated, dialyzed in a 3500 Da bag for 72 h, and freeze-dried. After these steps, the polysaccharides obtained through alkali solution degradation were designated as Al-Post-proc.

Acid solution for polysaccharides degradation: Hw was added into a 5 mg/mL solution using 0.1 M HCl solution. The solution was soaked and stirred for 3 h, after which the pH was adjusted to 7. Then, the solution was vacuum-concentrated, dialyzed in a 3500 Da bag for 72 h, and freeze-dried. After these steps, the polysaccharides obtained through acid solution degradation were designated as Ac-Post-proc.

### 2.3. The Composition of CTBPs

The carbohydrate content (%, *w*/*w*) was quantified using the phenol–sulfuric acid method, with d-glucose serving as the standard [20]. The protein content (%, *w*/*w*) was assessed via Bradford’s method, utilizing bovine serum albumin as the standard [21]. The total phenol content (%, *w*/*w*) was determined by the Folin–Ciocalteu method, using gallic acid as the standard [22]. The content of polysaccharide Glucuronic acid was determined by m-hydroxydiphenyl colorimetry, using Galacturonic acid as the standard [23].

The monosaccharide composition of CTBPs was analyzed by Ion Chromatography (ICS5000, ThermoFisher, Waltham, MA, USA). An accurate weight of 5 mg of the sample was put into ampoules with 2 mL of 3 M TFA, and hydrolyzed at 120 °C for 3 h. The acid hydrolysis solution was precisely drawn, then transferred to a tube and blown dry in a nitrogen environment, then 5 mL of water was added, and the resultant mixture was vortexed to mix thoroughly and centrifuged at 12,000× *g* rpm for 5 min. The supernatant was then transferred for IC analysis. The treatment of standard articles was the same as above. The chromatographic column used was a Dionex Carbopac^TM^ PA10 (046110, Waltham, MA, USA) (4 × 250 mm). The mobile phase consisted of A: H_2_O; B: 500 mM NaOH and 50 mM NaOAc; C: 20 mM NaOH. The flow rate was set at 1.0 mL/min, with an injection volume of 25 µL and column temperature maintained at 30 °C. The detector employed was an electrochemical detector (ICS5000, Waltham, MA, USA).

### 2.4. The Molecular Weight (Mw) of CTBPs

The Mw was determined using a high-performance size-exclusion chromatography (HPSEC) system equipped with a multi-angle laser light scattering detector (MALLS, DAWN HELEOS II, Wyatt Technology, Goleta, CA, USA) and a refractive index detector (Agilent G1362A, Santa Clara, CA, USA) [24]. Column: Ultrahydrogel 2000 SEC column (7.8 × 300 mm) in series; Ultrahydrogel 1000 SEC (7.8 × 300 mm); mobile phase: 0.1 mol/L sodium nitrate (containing 0.05% sodium azide) solution; flow rate: 0.6 mL/min; column temperature: 35 °C; injection volume: 100 µL.

### 2.5. The Particle Size and Zeta Potential of CTBPs

The particle size and zeta potential of CTBPs were detected using an a dynamic light scattering instrument (Zetasizer Nano S90, Malvern Instruments, Worcestershire, UK) [25]. The CTBPs were dissolved in deionized water for sample analysis.

### 2.6. Spectral Analysis of CTBPs

The CTBPs were added into a solution at a concentration of 0.1 mg/mL using distilled water, and scanned with an ultraviolet–visible spectrophotometer (UV-754, Precision Scientific Instruments Co., Ltd., Shanghai, China) over a wavelength range of 190–600 nm [26]. The infrared spectra of CTBPs were analyzed using the KBr pellet method. CTBPs and KBr powder were thoroughly ground and pressed into disks, which were then examined using the Fourier-transformed infrared spectrometer (Bruker Vector 33, Bruker, Germany) over a scanning range of 4000 to 400 cm⁻^1^ [26].

### 2.7. Congo Red Experiments

Referring to the previous method with some modifications [27], 1 mg/mL CTBPs and 1 mg/mL Congo red solution were prepared and mixed in equal volumes. Subsequently, varying volumes of NaOH solution were added to achieve final concentrations of 0, 0.1, 0.2, 0.3, 0.4, and 0.5 M. After allowing the mixture to stand for 30 min, the ultraviolet–visible absorption spectrum was scanned over a range of 400–600 nm to determine the maximum absorption wavelength.

### 2.8. Water Holding Capacity Analysis

Precisely measure 200 mg of CTBPs (W_0_) and transfer it to a pre-weighed 2 mL centrifuge tube (W_1_). Subsequently, introduce 1 mL of distilled water into the centrifuge tube, mix thoroughly, and allow the mixture to stand at room temperature for 2 h. Centrifuge the mixture at room temperature at 10,000 rpm for 15 min. Discard the supernatant, and weigh the remaining material (W_2_). The water holding capacity of CTBPs is determined using Formula (1).

(1) $$\mathrm{Water}\;\mathrm{holding}\;\mathrm{capacity}\;(g/g)=\frac{W_{2}-W_{1}-W_{0}}{W_{0}}$$

### 2.9. Oil Holding Capacity Analysis

Precisely weigh 200 mg of CTBPs (W_0_) and add it to a 2 mL centrifuge tube (W_1_). Subsequently, introduce 1 mL of edible oil into the centrifuge tube, mix thoroughly, and allow the mixture to stand at room temperature for 2 h. Centrifuge the mixture at room temperature at 10,000 rpm for 15 min. Discard the supernatant and weigh the remaining material (W_2_). The oil holding capacity of CTBPs is determined using Formula (2).

(2) $$\mathrm{Oil}\;\mathrm{holding}\;\mathrm{capacity}\;(g/g)=\frac{W_{2}-W_{1}-W_{0}}{W_{0}}$$

### 2.10. Bile Acid Binding Capacity of CTBPs

The experimental procedure was adapted from prior research [28]. Stock solution of sodium taurocholate was prepared in 0.1 mol/L phosphate-buffer solution (PBS) at a pH of 6.8 and subsequently diluted to concentrations of 0.05, 0.10, 0.15, 0.20, 0.25, and 0.30 mmol/L. The absorbance of each solution was measured at 387 nm following a pre-treatment protocol involving incubation of 1 mL samples in 60% (*w*/*w*) sulfuric acid at 70 °C for 20 min, followed by cooling in an ice bath for 5 min. The resulting linear regression equation was: y = 1.3048x + 0.122, R^2^ = 0.9973. CTBPs, porcine trypsin, and sodium taurocholate were dissolved in 0.1 M PBS (pH 6.8). Subsequently, 0.5 mL of a 5 mg/mL CTBPs solution was mixed with 0.5 mL of 0.01 M HCl solution and incubated in a constant-temperature water bath at 37 °C for 60 min. The pH was then adjusted to 6.8 using a 0.1 M NaOH solution. Next, 2 mL of a 10 mg/mL porcine trypsin solution and 2 mL of a 0.3 mM sodium taurocholate solution were added, mixed thoroughly, and the mixture was incubated at 37 °C for an additional 60 min. Finally, the reaction mixture was centrifuged at 4000× *g* for 20 min, and 2.5 mL of the resulting supernatant was combined with 7.5 mL of 60% (*w*/*w*) H_2_SO_4_ solution, heated at 70 °C for 20 min, and subsequently cooled in an ice-water bath for 5 min. The absorbance was measured at 387 nm. The bile acid-binding capacity of CTBPs was determined based on the standard curve of sodium taurocholate.

### 2.11. Activities Analysis

#### 2.11.1. α-Glucosidase Inhibitory Activities of CTBPs

The inhibitory effect of CTBPs on *α*-glucosidase was assessed according to a previously established method [16]. Specifically, 30 μL of CTBPs solution (5 mg/mL) or positive control (Acarbose hydrate) was incubated with 30 μL of *α*-glucosidase diluent at 37 °C for 10 min in 0.1 M PBS (pH 6.8). Subsequently, 30 μL of p-nitrophenyl-*α*-d-glucopyranoside (p-NPG) solution was added to the mixture. After thorough mixing, the reaction was continued at 37 °C for 15 min, followed by the addition of 0.1 mL of 0.1 M Na_2_CO_3_ solution to terminate the reaction. The absorbance was measured at 405 nm using a spectrophotometer. The inhibitory effect was quantified using the following Equation (3):

(3) $$\mathrm{Inhibition}\left(\%\right)=\left[1-\frac{\left(A_{\mathrm{test}}-A_{\mathrm{test}\;\mathrm{blank}}\right)}{\left(A_{\mathrm{control}}-A_{\mathrm{control}\;\mathrm{blank}}\right)}\right]\times 100\%$$

where A_test_ is the light absorption value of the CTBPs, *α*-glucosidase, and p-NPG reaction solution; A_test blank_ is the light absorption value of the CTBPs solution; A_control_ is the light absorption value of the *α*-glucosidase and p-NPG reaction solution; and A_control blank_ is the light absorption value of the buffer solution.

#### 2.11.2. Antioxidant Capacities of CTBPs

1,1-diphenyl-2-picrylhydrazyl (DPPH) free radical scavenging test: 2 mL CTBPs solution was mixed with 2 mL 0.2 mM DPPH ethanol solution, and the absorbance was measured at 517 nm after incubation at room temperature for 30 min. Distilled water was employed as a blank control, and the experiment was conducted in triplicate [17].

2,2′-azino-bis-(3-ethyl-benzothiazoline-6-sulfonic acid) (ABTS) free radical scavenging test: The ABTS working solution was prepared by mixing equal volumes of a 7 mM ABTS solution and a 2.45 mM potassium persulfate solution, followed by incubation at room temperature for 12 h in the dark. Before use, the ABTS working solution was diluted with PBS (pH 7.4) to achieve an absorbance of 0.70 ± 0.02 at 734 nm. A total of 40 μL of CTBPs solution was added to 3960 μL of ABTS working solution, mixed thoroughly, and allowed to react for 6 min at room temperature in the dark. The absorbance was measured at 734 nm. Distilled water served as the blank control and the experiment was conducted in triplicate [17].

#### 2.11.3. Anti-Glycation Activities of CTBPs

The anti-glycation activities of CTBPs were evaluated using a glycation model based on bovine serum albumin (BSA). Specifically, BSA, fructose, and CTBPs were dissolved in 0.1 M PBS (pH 7.4, containing 0.02% sodium azide to prevent microbial contamination). The BSA and fructose solutions were thoroughly mixed, and CTBPs were added to achieve final concentrations of 20 mg/mL for BSA, 90.1 mg/mL for fructose, and 0.3 mg/mL for CTBPs. After mixing, the solution was incubated at 50 °C for 24 h in a water bath. Following rapid cooling in an ice bath, the glycosylated BSA model was prepared [29].

The inhibitory effect of the extract on fructosamine formation in glycosylated products was evaluated using the nitro blue tetrazolium chloride (NBT) method [30]. A total of 100 μL of glycosylated protein solution and 200 μL of NBT solution (0.3 M, dissolved in 0.1 M carbonate buffer, pH 10.35) were mixed, and incubated at room temperature in the dark for 30 min. Absorbance was measured at 530 nm. The inhibition rate was determined by comparing the absorbance values of samples with and without added polysaccharides.

The rate of dimethyl formation inhibition was assessed using Girard-T reagent [31], which is a derivatizing agent for carbonyl compounds and forms stable hydrazones. Girard-T reagent was dissolved in 0.5 M pH 2.9 sodium formate solution, and the Girard-T reagent was mixed with the glycosylated BSA protein solution in equal volume, and the reaction was carried out at room temperature for 1 h, away from light. The absorption value of the solution was measured at 290 nm. The inhibition rate was determined by comparing the absorbance values of samples with and without added polysaccharides.

The inhibitory effect of extracts on advanced glycation end-product (AGE) generation was assessed using the method described by Spínola [32]. The fluorescence intensity of the BSA protein solution after 60 μL glycoylation was uniformly mixed with 2 mL of phosphate buffer (0.2 M, pH 7.4) at an excitation wavelength of 370 nm and an emission wavelength of 440 nm. The inhibition rate was calculated according to the fluorescence intensity before and after adding polysaccharide.

The inhibitory effect of the extract on protein oxidation products was assessed by measuring the fluorescence intensity of the protein system at excitation/emission wavelengths of 330/415 nm, 365/480 nm, and 325/434 nm [33].

### 2.12. Statistical Analysis

Data were analyzed using SPSS (version 29.0); differences were assessed via one-way ANOVA followed by Duncan’s test and post hoc tests. Results are presented as mean ± SD.

## 3. Results and Discussion

### 3.1. Yields and Composition of CTBPs

Different preparation methods significantly influence the yield and composition of CTBPs, as illustrated in Table 1. Compared to the traditional hot water extraction method, both alkali solution extraction and ultrasonic-assisted extraction methods have shown enhanced polysaccharide yields. Specifically, the yield of Al reached the highest level at 7.72 ± 0.09%, while the yield of Ac was the lowest at 4.26 ± 0.16%. Moreover, Ac exhibited the highest uronic acid content (13.07 ± 0.13%). These findings were in line with the extraction results of polysaccharides from blackberry [34]. When various solvents were employed for blackberry polysaccharide extraction, the highest yield was achieved using alkaline solutions, and the lowest yield was observed with acid solutions (only 2.88%). The higher extraction rate achieved with alkali solutions can be attributed to their ability to disrupt plant cell walls [35], thereby facilitating the effective release of plant polysaccharides [36]. Conversely, the lower extraction rate observed with acid solutions is primarily due to the degradation of plant polysaccharides by the acid. This degradation reduces the molecular weight of the polysaccharides, converting them into smaller molecules, and even free sugars. During subsequent processing steps, such as alcohol precipitation and dialysis, these smaller molecules and free sugars are removed, leading to a significant decrease in the overall extraction yield of polysaccharides [15].

Even after undergoing alcohol precipitation and dialysis, trace amounts of small molecules, such as phenols and proteins, may remain. As shown in Table 1, the CTBPs exhibited varying contents of compounds, with carbohydrate content ranging from 53.86 ± 0.52% to 78.79 ± 0.62%, total phenol content from 70.60 ± 1.31 to 81.69 ± 0.70 mg GAE/g, and protein content from 3.83 ± 0.05% to 4.82 ± 0.10%. Among them, Al had the highest content of carbohydrates (78.79 ± 0.62%), total phenol (81.69 ± 0.70 mg GAE/g), and protein (4.82 ± 0.10%). The high content of various compounds in Al can be attributed to the effective disruption of the cell wall of the plant material by the alkali solution, which facilitates the dissolution of polysaccharides. This finding is consistent with the results of alkaline extraction of polysaccharides from other sources, such as *Clitocybe squamulosa* [26]. The use of alkali solution enhances extraction efficiency by breaking down the cell wall structure, allowing for better release and recovery of the target compounds.

### 3.2. Monosaccharide Composition and Molecular Weight

**Figure 2:**
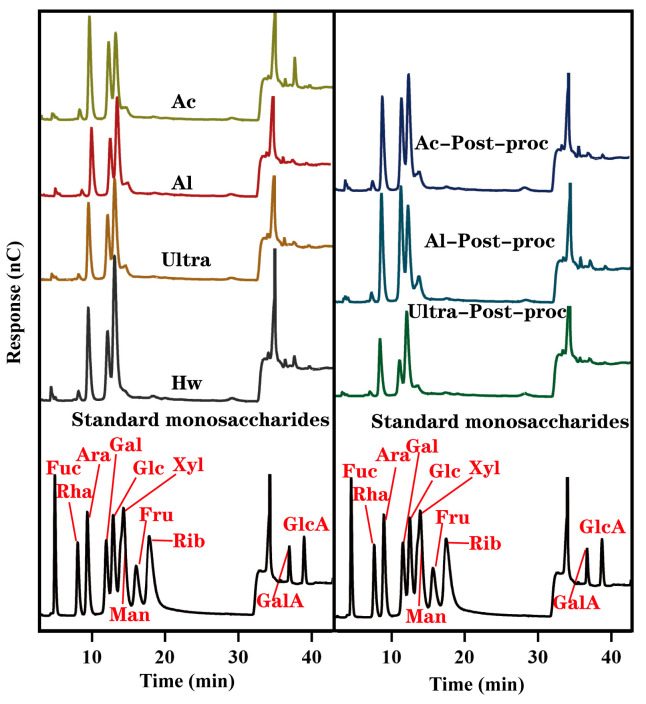
Ion chromatogram of CTBPs and standard monosaccharides.

As the fundamental building block of polysaccharides, monosaccharide composition is critical for elucidating their structural characteristics and determining their biological activities [37]. The ion chromatogram of CTBPs, and the monosaccharide composition of each component, was calculated after retention time and response strength, as shown in Figure 2. As demonstrated in Table 1, CTBPs consisted of Fucose, Rhamnose, Arabinose, Galactose, Glucose, Mannose, Xylose, Galacturonic acid and Glucuronic acid composition. Arabinose, Galactose and Glucose accounted for the most monosaccharides in CTBPs, ranging from 26.69% to 28.76%, 19.58% to 32.78%, and 20.47% to 36.00%, respectively. In addition, CTBPs also contained Galacturonic acid and Glucuronic acid, indicating that CTBPs were acidic polysaccharides. The Galacturonic acid content in Ac was the highest, which was consistent with the results of blackberry polysaccharides extracted from the acid solution [34]. The results of the monosaccharide experiment showed that different methods of polysaccharide acquisition would affect the proportion of monosaccharide composition, but would not change the composition type of the monosaccharide.

The Mw of polysaccharides is closely related to the extraction method used, and the biological activity of polysaccharides varies with their molecular weight. As shown in Table 1, the Mw of CTBPs were as follows: Hw (6.62 × 10^5^ Da) > Al (4.01 × 10^5^ Da) > Ac-Post-proc (2.26 × 10^5^ Da) > Ac (1.95 × 10^5^ Da) > Ultra (1.77 × 10^5^ Da) > Al-Post-proc (1.69 × 10^5^ Da) > Ultra-Post-proc (1.45 × 10^5^ Da), in descending order. The Hw group demonstrated the highest Mw among the analyzed samples, consistent with prior observations in banana flower polysaccharide extraction studies, that polysaccharides extracted through hot water, alkaline, acid, and ultrasound-assisted methods exhibit a progressive decline in Mw [38]. In the present study, similar extraction and degradation techniques were applied to CTBPs, revealing a comparable trend of Mw reduction. These findings indicate that the selection of extraction methodologies can significantly influence the Mw of polysaccharides, enabling the targeted isolation of low-molecular-weight variants through optimized protocols. This approach provides a strategic pathway for obtaining polysaccharides with specific molecular characteristics.

### 3.3. Particle Size and Zeta Potential Analysis

**Figure 3:**
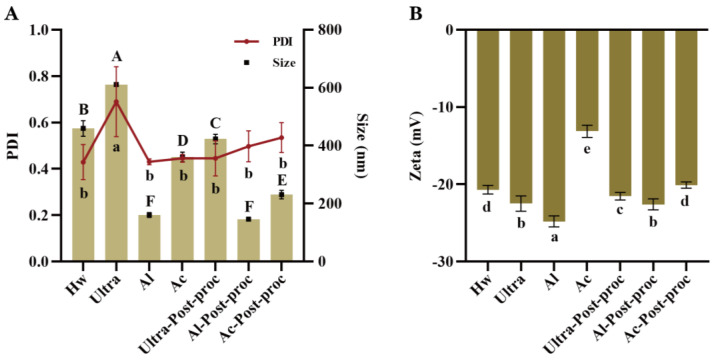
Particle size (**A**) and zeta potential (**B**) of CTBPs. Different letters for the same indicator indicate significant differences, *p* < 0.05.

The particle size and polydispersity index (PDI) coefficient serve as critical indicators of polysaccharide dimensions and their distribution homogeneity in a solution. As illustrated in Figure 3A, CTBPs exhibited a particle size distribution spanning 146.57 to 610.93 nm. Notably, Al-Post-proc and Al demonstrate the smallest particle sizes, at 146.56 nm and 160.67 nm, respectively. Furthermore, Al displayed the lowest polydispersity index (PDI) value, suggesting superior uniformity in its aqueous dispersion. Figure 3B revealed that Al and Al-Post-proc exhibited significantly elevated zeta potential magnitudes, measuring 25.17 mV and 22.73 mV, respectively, surpassing those observed in other experimental groups. This enhanced electrostatic potential directly correlates with solution stability, as systems with greater absolute zeta potential values demonstrate increased resistance to particle aggregation [39]. It can be seen that the smaller the particle size, the more stable the polysaccharide solution, and the better the application in food and medicine.

### 3.4. Spectral Analysis

**Figure 4:**
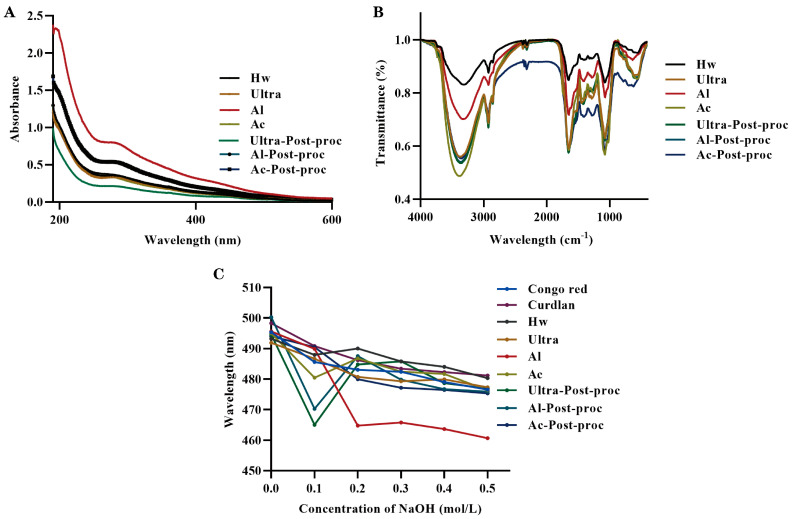
The ultraviolet spectra (**A**), FT-IR spectra (**B**), and Congo red experiment results (**C**) of CTBPs.

Ultraviolet (UV) scans can detect the presence of proteins in polysaccharide samples by identifying specific absorption peaks. Figure 4A illustrated the UV scanning results of CTBPs; the absorption peaks at 280 nm varied in intensity among the seven UV scanning curves. This variation correlated with the protein content data presented in Table 1, confirming that each component of the polysaccharide contained a small amount of protein.

Fourier transform infrared spectroscopy (FT-IR) was used to analyze the characteristic functional groups of CTBPs. As shown in Figure 4B, the characteristic absorption peaks of polysaccharides appeared in the infrared spectra between 4000 and 400 cm^−1^. The infrared spectra of the seven polysaccharides were similar, and the absorption peaks did not shift significantly. Specifically, CTBPs mainly exhibited vibration absorption peaks at 3358, 2922, 1652, 1411, 1284, 1074, and 877 cm^−1^. The strong absorption peak at 3358 cm⁻^1^ was caused by the stretching vibration of O-H bonds, and the absorption peak at 2922 cm^−1^ was caused by the asymmetric stretching vibration of C-H bonds [40]. The absorption peak at 1652 cm^−1^ was characteristic of bound water [41]. The absorption peak at 1411 cm^−1^ was due to the variable angle vibration of C-H or O-H bonds [42]. The absorption peaks at 1284 and 1074 cm^−1^ indicated the presence of C-O-C and C-O-H bonds [34]. Additionally, the absorption peak at 877 cm^−1^ in the infrared spectrogram of the polysaccharide was attributed to the presence of *β*-glucoside bond [43].

### 3.5. Conformational Analysis

The interaction between the Congo red solution and polysaccharides containing a triple helix structure resulted in a redshift of the maximum absorption wavelength of the solution [44]. As illustrated in Figure 4C, when compared to pure Congo red solution, only the Al-Post-proc exhibited a noticeable redshift in wavelength. This observation indicated that Al-Post-proc was the only polysaccharide among those tested that possessed a triple helix conformation. For the other polysaccharides, no significant redshift was observed in their maximum absorption peaks, and there was no clear correlation with increasing NaOH concentration. It has been found that the stability of both intramolecular and intermolecular hydrogen bonds affects the triple helix structure of polysaccharides [26]. Specifically, the disruption of these hydrogen bonds leads to the destruction of the triple helix structure. The observed differences in the redshift of CTBPs can be attributed to variations in hydrogen bond content and sample composition [45].

### 3.6. Water and Oil Holding Capacity

**Figure 5:**
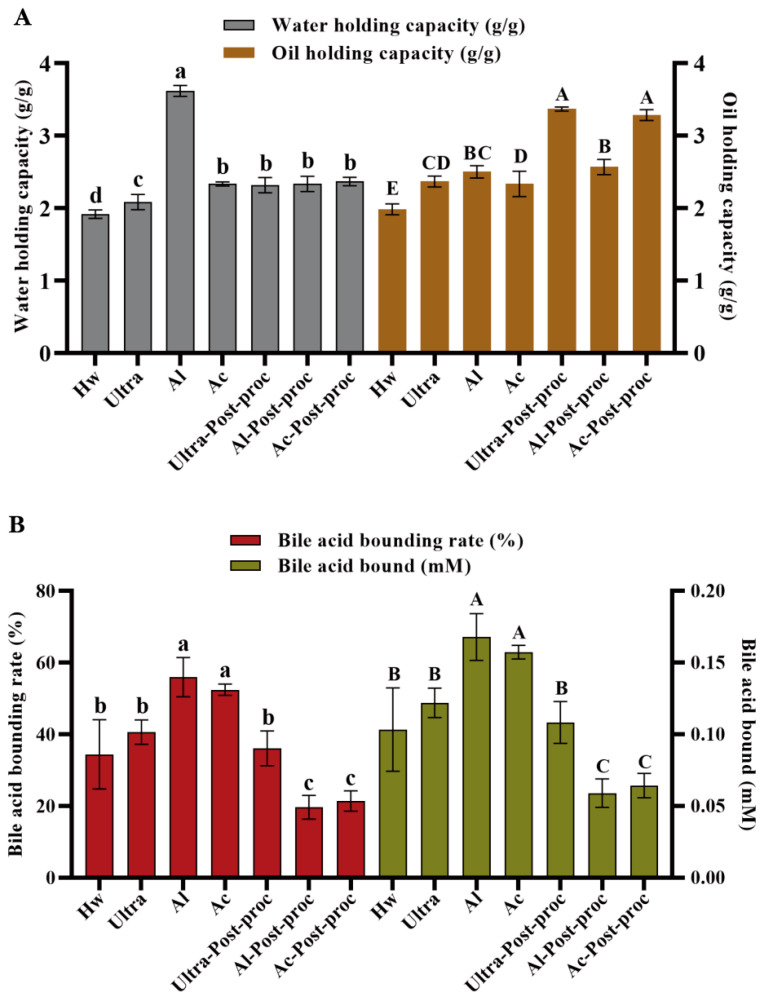
Water and oil holding capacity (**A**) and bile acid-binding ability (**B**) of CTBPs. Different letters for the same indicator indicate significant differences, *p* < 0.05.

As depicted in Figure 5A, the CTBPs exhibited superior water- and oil-holding properties. Al demonstrated the highest water binding capacity, reaching 3.61 ± 0.08 g/g. This elevated capacity could be ascribed to smaller particle sizes, which afforded a greater surface area for contact, thereby enhancing its water absorption capability. The stability of the polysaccharides, as assessed by their water holding properties, was consistent with the zeta potential results observed for Al. The oil binding capacities of Ultra-Post-proc and Ac-Post-proc were measured at 3.37 ± 0.03 g/g and 3.28 ± 0.08 g/g, respectively. Polysaccharides with high oil-binding abilities could be utilized to preserve food flavor and serve as functional food components to regulate body weight and blood lipid levels. They could also reduce the absorption of dietary fats in the intestine. These functional properties might be attributed to the lower molecular weights and smaller particle sizes of the treated polysaccharides, which permitted greater contact with oil and enhanced physical trapping capabilities.

### 3.7. Bile Acid Binding Ability

The liver converts cholesterol into bile acids, and the addition of polysaccharides facilitates the excretion of cholesterol through the intestine, thereby reducing plasma cholesterol levels [34]. Previous studies have demonstrated that polysaccharides possess the ability to bind bile acids, and the binding capacity of blackberry polysaccharides to bile acids decreases as their Mw decreases [34]. However, a similar trend was not observed in this experiment, which can be attributed to the influence of different extraction and degradation methods on the properties of the polysaccharides, rather than changes in Mw. As illustrated in Figure 5B, no significant difference was observed in the bile acid binding capacity between Al and Ac. Specifically, the bile acid binding capacity of Al was 0.17 ± 0.02 mM, with an adsorption rate of 55.97 ± 5.43%.

### 3.8. Biological Activities

#### 3.8.1. α-Glucosidase Inhibitory Activity

**Figure 6:**
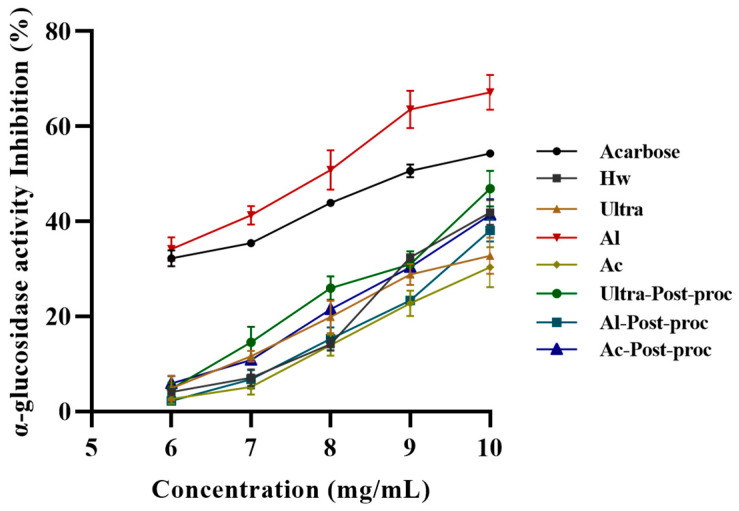
*α*-glucosidase inhibitory activity of CTBPs.

*α*-glucosidase, an essential enzyme in carbohydrate digestion, accelerates Glucose production, leading to elevated blood sugar levels [46]. Therefore, identifying natural substances that can inhibit *α*-glucosidase activity is crucial. It is known that almost all polysaccharides (especially in high concentration) show inhibition of α-glucosidase. As depicted in Figure 6, the inhibitory effects of CTBPs and Acarbose (the positive control) on *α*-glucosidase were experimentally evaluated. The results indicated that both CTBPs and Acarbose exhibited dose-dependent inhibition of *α*-glucosidase, with Al (IC_50_ 7.71 mg/mL) demonstrating superior inhibitory activity compared to Acarbose. These findings suggest that CTBPs, particularly Al, have the potential to be developed as effective *α*-glucosidase inhibitors. This could be attributed to the fact that the alkali solution can destroy the plant cell wall [34], allowing more active substances to dissolve out; corresponding to the measured chemical composition results, Al contains more active substances.

#### 3.8.2. Antioxidant Activities

**Figure 7:**
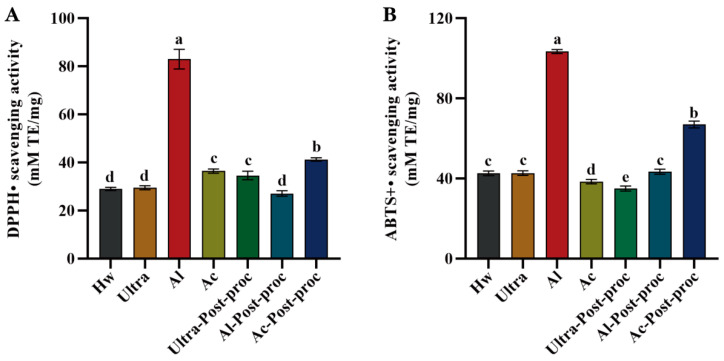
Antioxidant activities of CTBPs: DPPH radical scavenging activity (**A**); ABTS radical scavenging activity (**B**). Different letters for the same indicator indicate significant differences, *p* < 0.05.

Oxidative stress is associated with chronic diseases such as aging, diabetes, and cardiovascular disease. Many natural compounds in nature act as antioxidants, scavenging free radicals and reducing oxidative stress. Plant polysaccharides have been proven to be effective antioxidants with strong free radical scavenging abilities [47]. As depicted in Figure 7A, the scavenging ability of CTBPs (Chrysanthemum polysaccharides) for DPPH free radicals follows the following order: Al > Ac-post-proc > Ac ≈ Ultra-post-proc > Hw ≈ Ultra ≈ Al-post-proc, with Al demonstrating the strongest scavenging ability at 82.96 ± 4.11 (mM TE/mg). ABTS is a widely used reagent for the detection of the antioxidant capacity of substances in vitro. Consistent with the DPPH results, Al also exhibited the highest scavenging power for ABTS free radicals, as shown in Figure 7B, with a value of 103.37 ± 0.99 (mM TE/mg). These findings indicate that polysaccharides extracted using alkaline solutions exhibit superior antioxidant activity. This observation is consistent with the antioxidant results of *Sargassum* polysaccharides [16], and can be attributed to the higher total phenol content of alkaline-extracted polysaccharides compared to other groups.

#### 3.8.3. Anti-Glycation Activities

**Figure 8:**
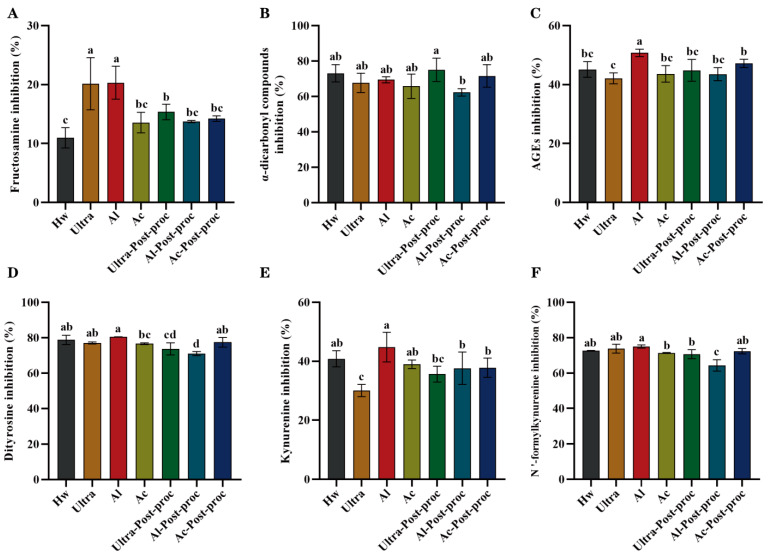
Inhibitory rate of CTBPs on glycosylation products: fructosamine (**A**); *α*-dicarbonyl compound (**B**); AGEs (**C**); dityrosine (**D**); kynurenine (**E**); N’-formylcanurine (**F**). Different letters for the same indicator indicate significant differences, *p* < 0.05.

Glycation is a non-enzymatic reaction where sugars react with proteins, lipids, and other macromolecules to produce irreversible advanced glycation end products (AGEs). The accumulation of AGEs in the body can lead to a variety of health problems, affecting multiple systems including the skin, eyes, cardiovascular system, and nervous system. In severe cases, this accumulation can result in fatal diseases. Therefore, controlling sugar intake and maintaining a healthy lifestyle are crucial measures to prevent glycation-related diseases [48]. As depicted in Figure 8A, CTBPs exhibited effective inhibition of fructosamine. Specifically, the inhibitory effect of Ultra and Al was the strongest, which showed significant differences compared to other groups (*p* < 0.05). During the intermediate stage of glycation, *α*-dicarbonyl compounds are generated. As shown in Figure 8B, the inhibition rates of CTBPs on these intermediate products were high across all groups, with minimal differences between groups. However, in the inhibition of AGEs, as illustrated in Figure 8C, Al exhibited the most prominent activity, indicating that its inhibitory effect on glycation was stronger in the final stage compared to the initial stages, with a significant difference compared to the other groups (*p* < 0.05). In general, the inhibitory effect of CTBPs on glycosylated products was different from that of polysaccharides with higher Mw, as reported by Sun et al. [49], which showed better inhibitory activities on glycosylated products, but that of Al with medium Mw showed better activities.

Non-enzymatic glycation often occurs concurrently with oxidation, during which tryptophan and tyrosine residues in proteins are oxidatively destroyed, leading to the formation of protein oxidation markers with distinct fluorescence characteristics. Specifically, tryptophan residues can be modified into dityrosine and N’-formylcanurine, while tyrosine residues can be converted into kynurenine [50]. It has been reported that polysaccharides can inhibit the formation of glycosylation and protein oxidation products [16,34]. As depicted in Figure 8D–F, CTBPs effectively inhibited the formation of protein oxidation products. Specifically, Al demonstrated a strong inhibitory effect on the formation of dityrosine, kynurenine, and N’-formylcanurine (*p* < 0.05). By protecting tryptophan and tyrosine residues in proteins, Al can effectively mitigate the impact of glycosylation, thereby achieving an anti-glycosylation effect. Consistent with the results of glycation product inhibition, Al exhibited excellent inhibitory activity across all stages of glycation. This finding suggests that Al extracted by alkaline solution, with their moderate Mw, exposed and retained more active groups, contributing to their enhanced activities. These findings indicate that CTBPs has potential in anti-glycosylation drugs in the field of medicine.

## 4. Conclusions

In this study, CTBPs were prepared by different methods, and the chemical compositions, physicochemical properties, and biological activities of these polysaccharides were compared. CTBPs were composed of Arabinose, Galactose, and Glucose. The yield of Al reached the highest level, at 7.72 ± 0.09%, while the yield of Ac was the lowest, at 4.26 ± 0.16%. The contents of carbohydrates, total phenol, and protein in Al were the highest, at 78.79 ± 0.62%, 81.69 ± 0.70 mg GAE/g, and 4.82 ± 0.10%, respectively. The uronic acid content of Ac exhibited the highest level at 13.07 ± 0.13%. Compared with traditional hot water extraction, the Mw of CTBPs obtained by the other six methods was reduced to different degrees. CTBPs contained typical polysaccharide characteristic functional groups, among which Al-Post-proc had a triple helix structure. In addition, CTBPs also had good water-holding capacity, oil-holding capacity, and bile acid-binding capacity. In vitro experiments showed that Al possessed the best *α*-glucosidase inhibitory activity, antioxidant activity, and anti-glycosylation activity. Our findings can provide a theoretical basis for the development and utilization of CTBPs as natural active substances, and have potential applications in pharmaceuticals or functional foods.

## Figures and Tables

**Table 1:** Chemical composition of CTBPs (polysaccharides of *Coreopsis tinctoria* buds).

Sample	Hw	Ultra	Al	Ac	Ultra-Post-Proc	Al-Post-Proc	Ac-Post-Proc
Yield (%)	5.51 ± 0.16 ^c^	6.42 ± 0.10 ^b^	7.72 ± 0.09 ^a^	4.26 ± 0.16 ^d^	-	-	-
Carbohydrate (%)	64.96 ± 0.65 ^e^	67.47 ± 0.57 ^d^	78.79 ± 0.62 ^a^	76.37 ± 0.68 ^b^	53.86 ± 0.52 ^f^	64.74 ± 0.65 ^e^	71.87 ± 0.39 ^c^
Total phenol (mg GAE/g)	75.74 ± 0.14 ^d^	76.74 ± 0.16 ^c^	81.69 ± 0.70 ^a^	70.60 ± 1.31 ^e^	76.98 ± 0.21 ^c^	78.33 ± 0.43 ^b^	78.85 ± 0.40 ^b^
Protein (%)	4.20 ± 0.06 ^d^	4.40 ± 0.08 ^c^	4.82 ± 0.10 ^a^	4.72 ± 0.02 ^b^	3.94 ± 0.02 ^e^	3.88 ± 0.02 ^ef^	3.83 ± 0.05 ^f^
Uronic acid	7.27 ± 0.04 ^f^	8.26 ± 0.02 ^d^	10.41 ± 0.07 ^b^	13.07 ± 0.13 ^a^	6.99 ± 0.02 ^g^	7.79 ± 0.09 ^e^	8.84 ± 0.23 ^c^
Monosaccharide composition (%)							
Fucose	0.71	0.59	0.64	0.58	0.68	0.82	0.67
Rhamnose	2.77	2.02	2.42	3.04	2.23	2.47	2.71
Arabinose	26.81	28.10	26.69	28.18	28.76	27.53	26.87
Galactose	22.07	26.15	25.05	23.69	19.58	32.78	29.07
Glucose	35.07	31.44	32.86	20.47	36.00	20.58	28.11
Mannose	2.79	3.15	3.80	3.20	3.18	4.28	3.96
Xylose	1.48	4.00	4.26	2.40	4.01	5.34	4.49
Galacturonic acid	7.13	3.29	3.14	17.34	4.54	4.89	2.68
Glucuronic acid	1.15	1.26	1.14	1.10	1.01	1.31	1.44
Molecular weight (kDa)							
Mw	6.62 × 10^5^	1.77 × 10^5^	4.01 × 10^5^	1.95 × 10^5^	1.45 × 10^5^	1.69 × 10^5^	2.26 × 10^5^
Mn	6.01 × 10^5^	6.98 × 10^4^	1.30 × 10^5^	7.48 × 10^4^	5.39 × 10^4^	6.74 × 10^4^	8.01 × 10^4^
Mw/Mn	1.10	2.54	3.11	2.61	2.68	2.51	2.82

Different letters under the same index indicate significant differences, *p* < 0.05. Mn: number-averaged molecular weight.

## Data Availability

The original contributions presented in the study are included in the article; further inquiries can be directed to the corresponding author.
